# Re-evaluation of EMG-torque relation in chronic stroke using linear electrode array EMG recordings

**DOI:** 10.1038/srep28957

**Published:** 2016-06-28

**Authors:** Minal Bhadane, Jie Liu, W. Zev Rymer, Ping Zhou, Sheng Li

**Affiliations:** 1 Department of Physical Medicine and Rehabilitation, University of Texas Health Science Center – Houston, Houston, TX, USA; 2 TIRR Memorial Hermann Research Center, Houston, TX, USA; 3 Sensory Motor Performance Program, Rehabilitation Institute of Chicago, Chicago, IL, USA; 4 Guangdong Work Injury Rehabilitation Center, Guangzhou, Guangdong Province, China

## Abstract

The objective was to re-evaluate the controversial reports of EMG-torque relation between impaired and non-impaired sides using linear electrode array EMG recordings. Ten subjects with chronic stroke performed a series of submaximal isometric elbow flexion tasks. A 20-channel linear array was used to record surface EMG of the biceps brachii muscles from both impaired and non-impaired sides. M-wave recordings for bilateral biceps brachii muscles were also made. Distribution of the slope of the EMG-torque relations for the individual channels showed a quasi-symmetrical “M” shaped pattern. The lowest value corresponded to the innervation zone (IZ) location. The highest value from the slope curve for each side was selected for comparison to minimize the effect of electrode placement and IZ asymmetry. The slope was greater on the impaired side in 4 of 10 subjects. There were a weak correlation between slope ratio and strength ratio and a moderate to high correlation between slope ratio and M-wave ratio between two sides. These findings suggest that the EMG-torque relations are likely mediated and influenced by multiple factors. Our findings emphasize the importance of electrode placement and suggest the primary role of peripheral adaptive changes in the EMG-torque relations in chronic stroke.

Weakness for voluntary muscle contraction is a common sequela of a hemispheric stroke. Together with other clinical symptoms, weakness is a primary contributor to the overall impairment^1^, specifically towards impaired motor control^2^. Loss of descending corticospinal pathway activation to spinal motoneurons after stroke is a direct consequence and is presumably a primary contributor to this weakness. Other pathological changes in the neuromuscular system may also contribute, such as differential loss of large motor units^3^,^4^, re-innervation of low-threshold motor units^4^,^5^,^6^, failure to increase motor unit firing rate during voluntary activation in paretic muscles^7^,^8^, and disuse atrophy, fat infiltration and contracture^9^,^10^,^11^. The failure to increase motor unit firing rate may be a maladaptive change as a result of the former, but may be related to other motor unit recruitment characteristics, such as reinnervation of low-threshold motor units and decreased motor unit recruitment thresholds.

These central and peripheral pathological changes profoundly affect the EMG-torque (force) relation in stroke survivors^12^. Two previous research studies^7^,^8^ have examined the EMG-torque relation during isometric elbow flexion torque generation at 90 degrees of flexion with matched torques. They found that the regression slope of the biceps brachii EMG and elbow joint torque relationship was significantly greater on the impaired side than on the contralateral side in approximately half of tested stroke subjects. The remaining group of stroke subjects showed smaller or similar slopes. The observation of greater slope on the impaired side was attributed to the requirement that these stroke subjects needed to recruit more high-threshold motor units in paretic muscles to produce the same amount of torque as compared with the non-impaired side (due to reduction of motor unit firing rates in paretic muscles). However the results of unchanged or smaller slopes in the other half of tested subjects were not discussed in detail. The controversial finding of altered slopes was also reported recently in a distal small muscle (first dorsal interosseus muscle) of stroke subjects^13^.

The mechanism underlying the relation between isometric muscle force and surface EMG amplitude is very complex. In addition to the aforementioned intrinsic pathological neuromuscular changes, there are other technical factors that could affect the EMG-torque relations, such as electrode placement^14^,^15^,^16^,^17^ and joint angle, i.e., muscle length^18^ and the contracting path, i.e., hyteresis^19^. Although these factors were taken into consideration in the previous studies^7^,^8^, the biceps brachii muscles may not be at the same length between the impaired and non-impaired side, given different levels of contracture and spasticity among stroke subjects (e.g., Ashworth scale range 1–5)^8^. Consequently, symmetrically located surface electrodes may not necessarily symmetrically record surface EMG signals in both impaired and non-impaired muscles, thus potentially contributing to controversial findings in tested stroke subjects.

To re-evaluate the EMG-torque relation in chronic stroke, we used a linear-electrode array to record surface EMG signals during isometric elbow flexion at different submaximal torque levels. The linear electrode array has dramatically expanded the applications of surface EMG in various areas^20^. The linear electrode array simultaneously records monopolar EMG signals from multiple channels that range from the proximal to distal end of the muscle longitudinally. From the location of innervation zone (IZ) where nerve terminations and muscle fibers are connected^21^,^22^; action potentials develop and propagate in opposite directions. Each channel of the array records surface EMG signals corresponding to a small area of muscle underneath the channel. Therefore, the EMG amplitude varies across individual single differential channels with the lowest value over the IZ location. As such, the slopes from different channels could reflect the effect of electrode placement and the IZ location on the EMG-torque relation. The evoked compound muscle action potential or M-wave (maximal muscle response to electrical nerve stimulation) amplitudes estimate the peripheral neuromuscular change, such as muscle fiber loss or atrophy. Comparison of the M-wave amplitudes between impaired and non-impaired biceps would reflect adaptive changes in the periphery.

In this study, we aimed to utilize the advantages of the linear electrode array EMG recordings to re-examine the EMG-torque relations in stroke subjects. Specific aims were to 1) compare IZ locations between impaired and non-impaired biceps muscles; 2) to investigate the effect of IZ location on the EMG-torque relation by comparing slopes of EMG-torque from individual channel EMG recordings; 3) to examine correlations among slopes, strength and M-wave amplitudes to investigate possible underlying pathophysiological mechanisms of motor impairment.

## Methods

### Subjects

In this study, we recruited 10 hemiparetic stroke survivors (age: 47–89 years; 7 male and 3 female). Table 1 displays the characteristics of stroke survivors. Inclusion criteria were: 1) hemiplegia secondary to an ischemic or hemorrhage stroke; 2) at least 6 months post-stroke; 3) spastic hypertonia in elbow flexors of the impaired side, rated as Modified Ashworth Scale (MAS) less than 3; 4) able to produce voluntary elbow flexion on the impaired side; and 5) able to follow instructions and give informed consent. Exclusion criteria included: 1) history of multiple strokes or bilateral involvement; 2) presence of contracture that would limit full elbow range of motion on the impaired side; 3) visual impairment including neglect. All subjects were recruited from outpatient clinic at TIRR Memorial Hermann Hospital in response to the recruitment flyer. All subjects were screened that they were stable medically and had no medication changes or adjustment in past 3 weeks. All subjects gave written informed consent prior to their participation. This study was approved by the Committee for the Protection of Human Subjects at The University of Texas Health Science Center at Houston and TIRR Memorial Hermann Hospital. The methods were carried out in accordance with the approved guidelines.

### Setup and data acquisition

**Figure 1:**
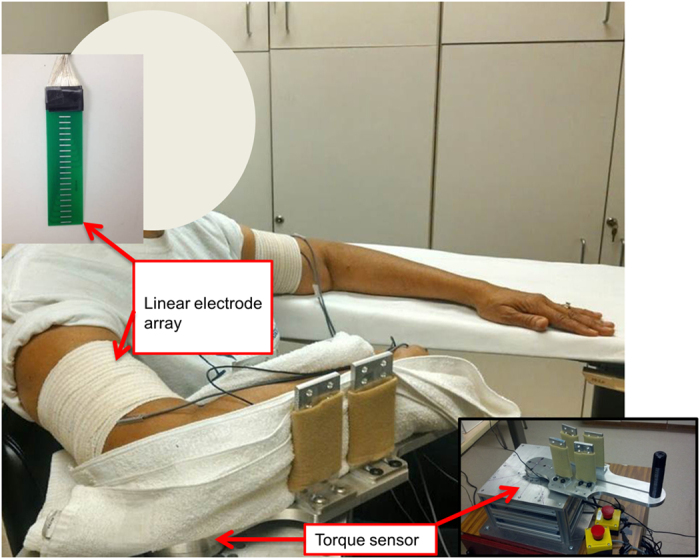
Customized experimental setup.

The subjects were seated on a height-adjustable chair. The arm to be tested was secured firmly on a customized apparatus (Fig. 1). The elbow joint was set to approximately 90° of flexion. The shoulder was positioned at approximately 45° of abduction and 30° of flexion. The hand and elbow were firmly secured using four vertical plates at the proximal and distal forearm. The center of the elbow joint was aligned with the axis of rotation of the shaft. This arrangement prevented translation and rotation of the arm. The other arm of the subject rested alongside the body. The subjects were explicitly instructed not to change or move the position of trunk during muscle contractions.

A customized linear electrode array was used for EMG recordings. The array has 20 silver bars (1mm width, 10 mm length) arranged in a linear manner with 5 mm distance between each bar. Target skin area was cleaned thoroughly with alcohol wipes. After standard skin preparation the electrode array was placed from the proximal to distal portion of the biceps brachii muscle with the midline of the array electrode aligned with the midline of biceps longitudinally from the biciptal groove to the biceps tendon insertion. As such, the electrode array covered the main portion of the muscle (Fig. 1). The same limb configuration and the electrode placement were used for the contralateral limb to ensure symmetrical limb configuration and electrode placement. The linear array was connected to the Porti system (TMS International, The Netherlands, sampling frequency 2000 Hz/channel, bandwidth: 10–500 Hz) to record 20-channel surface EMG signals. The reference electrode for both subject groups was attached to the lateral condyle of the humerus of the test arm. The electrodes were secured using self-adhesive tape to ensure contact.

Torque signals were recorded with a torque sensor (Model TRS 500, Transducers Techniques, CA), digitized at 1,000Hz on a PC computer with a BNC-2090A data acquisition board (National Instruments, Austin TX) using custom LabView software (National Instruments, Austin TX). Both torque and surface EMG recordings were triggered simultaneously. Data was saved for offline analysis using a customized MATLAB (The MathWorks Inc., Massachusetts, USA) program.

### Tasks

Subjects performed isometric elbow flexion tasks on each side. The order of side was randomized and balanced among subjects. Maximum voluntary contraction (MVC) of elbow flexion was first determined for each arm at approximately 90° of flexion after a few bouts of elbow flexion for warming up. Subjects were verbally encouraged to flex the elbow as hard as they can against the mechanical stops for 5 seconds (Fig. 1). The higher value among two MVC trials was accepted as the MVC value. Visual targets of 10, 20, 30, 40, 50, 60, 70, and 80% of MVC were established. Before a trial began, a visual target as a horizontal red line corresponding to one of 8 submaximal force levels was displayed on the computer screen. Subjects were verbally cued for the beginning of a 10 s trial. After a trial began, a real-time display of force signal as a white line ran from left to right. Subjects were instructed to initiate elbow flexion in a self-paced manner to reach the target after first tone (at 2 s) and stop after second tone (at 8 s). Subjects were verbally encouraged to match the force signal with the visual target as accurately as possible during all the trials, i.e., to match the white line (force signal) with the red line (target). Approximately 3~5 practice trials were allowed for all subjects to familiarize themselves with the task requirement. All sub-maximal isometric contractions were performed twice. Exactly same procedure was repeated on the other arm. In total the subjects performed 18 trials (2 repetitions of MVC trials and 2 repetitions of the 8 different targets) +3~5 practice trails for each arm. There was a required 2-minute break between MVC trials. Between each trial at submaximal levels, subjects were allowed to have sufficient rest to minimize possible muscle fatigue at their own pace.

The M-wave from both biceps muscles was recorded to estimate the peripheral neuromuscular capacity. We adopted a protocol for the M-wave measurement from our recent study^23^. In the described configuration, electrical stimulation was delivered to the musculocutaneous nerve using an electrical stimulator (D7SA, Digitimer Ltd, Hertfordshire, England). Electrical stimulation was triggered manually. The maximum response (M-wave) from the biceps muscle was detected when a higher intensity of electrical stimulation did not cause further increase in the on-line EMG response. After removing the stimulation artifacts^24^, the M-wave value was selected from the channel with the highest peak-to-peak value. We were not able to successfully remove the artifacts in one of stroke subjects. This subject was removed from M-wave analysis and correlation analysis.

### Data processing

**Figure 2:**
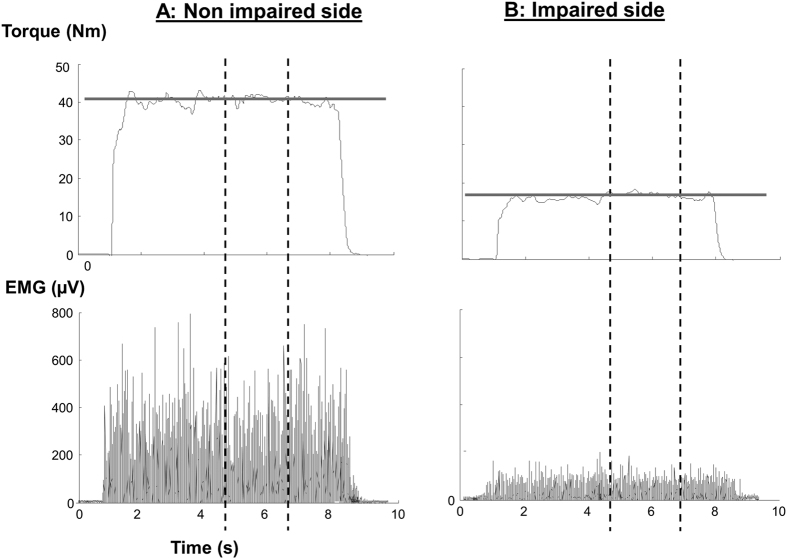
Representative Torque and rectified EMG data of a stroke subject at 80% MVC on both sides. Two vertical lines indicate a 2-second window of stable force output. Gray horizontal lines indicate the visual target displayed on the computer.

The torque signal was digitally filtered using a zero lag low-pass, second-order Butterworth filter with a 10Hz cutoff. EMG signals were filtered using a fourth-order band-pass (20–450Hz) Butterworth filter and a 60Hz notch filter. Consistent flat response of the torque data was observed for at least 4 seconds across all trials and subjects (see representative trials in Fig. 2). After torque was stabilized (smallest variation of torque signal) using visual inspection, a best segment of 2-second data (between two vertical lines in Fig. 2) was selected for further analysis, as in our previous studies^2^,^25^. EMG signals from the corresponding time window were selected for the data analysis. Data from 20 monopolar bars was processed by subtracting each pair of adjacent bars along the muscle fibers creating 19 single differential or bipolar channels. The exclusion criteria for the channels were 1) The channels close to the proximal or distal tendons; 2) the amplitude of the proximal/distal channels was too small or if there was a visibly large signal variation due to motion artifact. The average torque was calculated over selected segment while the EMG amplitude was calculated as the root mean square (RMS) value for individual channels.

**Figure 3:**
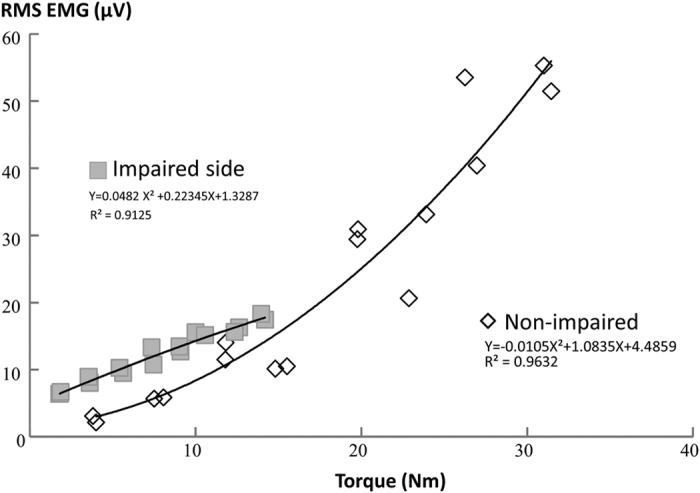
A representative EMG-torque relation on impaired and non-impaired sides in a stroke subject. The relation from the channel with the highest EMG values was selected. The figure was plotted from EMG and torque values for each repetition (x2 rep) at each target level (x8, from 10–80% MVC) for each side. There were 16 data points for each side. However, some data points were missing because some repetitions generated the same or very close EMG/Torque values.

**Figure 4:**
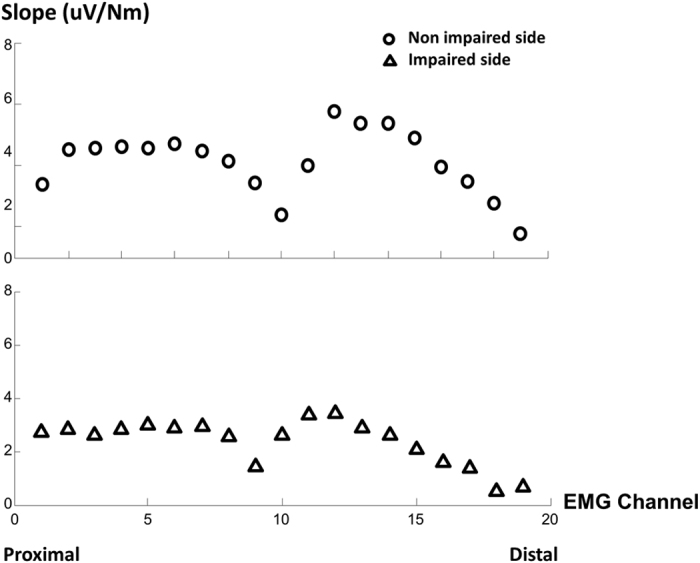
The “M” shaped distribution of slopes with the lowest slope over the IZ channel. Slopes are obtained from linear regressions between individual channel EMG and torque from a stroke subject.

EMG-torque relations were first established using EMG values from individual channels. Linear regression on EMG-torque relations was performed (representative subject data shown in Fig. 3). Slope and coefficient of determination (*R*^2^) were then obtained. Correlation coefficient (r) was then calculated from R^2^. Similarly, slopes of the EMG-torque relation for each channel were calculated for each subject. r for all subjects ranged from 0.81 to 0.99. Since the average torque value over selected window remained the same for any given trial across EMG channels, the slope of EMG-torque relations can then be considered to correspond to EMG activities across different channels from linear array recordings. The slope for each channel was presented in the ‘Slope Curve’ in Fig. 4. The lowest (over IZ) and highest points from the slope curve were calculated for both impaired and non-impaired sides. The ratio between the non-impaired and the impaired side was calculated for strength and the highest slope, respectively. As in Fig. 3, there was a curvilinear EMG-torque relation for the full range of tested torques with a smaller slope at low torques and a greater slope at high torques on the non-impaired side, while there was a linear EMG-torque relation on the impaired side. Due to the nonlinear EMG-force relation of biceps over a full range as reported in literature^7^,^8^, we only performed linear regression for matched range of tested torques on both sides.

### Detection of innervation zone (IZ) location

**Figure 5:**
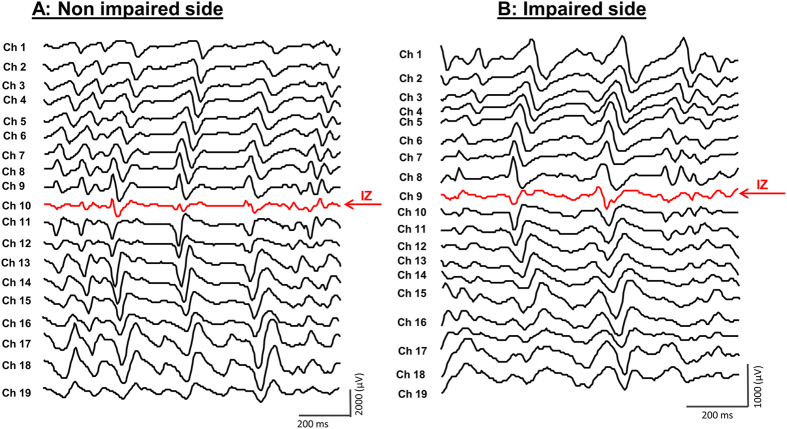
The innervation zone (IZ) channel location on the non-impaired (A) and impaired (B) side from a stroke subject.

The visual inspection of the bipolar signals was chosen for detection of IZ location rather than automated methods for better accuracy^26^. This was implemented for all trials across all stroke subjects. The criteria used for visually determining the IZ location were as follows (Fig. 5): (1) reversal in signal polarity in two adjacent channels; (2) a clear pattern of bidirectional action potential propagation from the IZ channel; (3) the lowest amplitude found over the IZ channel. Average of IZ location channel number from two trials at each MVC level was measured for further analysis. The mean IZ location was calculated by averaging IZ locations across different levels of activation.

### Statistics

Descriptive statistics were performed. To compare the IZ location between two sides and across different levels of activation, a two-way repeated-measures ANOVA was performed with the factors of SIDE (x2, impaired/non-impaired side) and ACTIVATION (x8 levels of activation, 10 to 80% MVC) to compare the IZ location. Highest and lowest points on the slope curve were compared between two sides by using a two-way ANOVA with factors of SIDE and SLOPE (x2, highest and lowest). Linear regression between strength ratio and slope ratio between two sides was performed. Linear regression between M-wave ratio and slope ratio between two sides was also performed. Our effect sizes were obtained from our preliminary trials, and were set to be 1.22 and 0.82 for slope and size (accounting for 60% and 40% of the variance respectively). Accordingly, statistical power was determined to be at least 80%. p < 0.05 was chosen to indicate statistically significant differences.

## Results

### EMG-Torque relations

**Figure 6:**
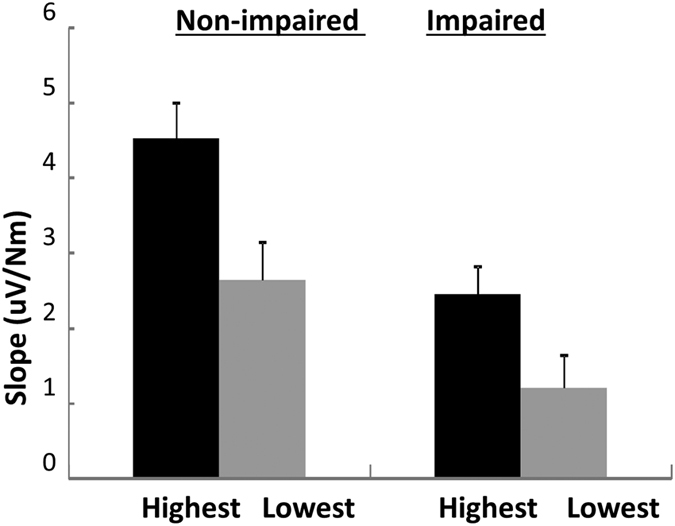
Mean slope obtained from highest and lowest (over innervation zone, IZ) individual channel EMG in stroke subjects. Standard errors are shown.

We first examined EMG-torque relations using EMG recordings from individual channels of the linear array. Distribution of slopes from individual channel EMG-torque relations demonstrated a unique pattern. As shown in Fig. 4, a quasi-symmetrical loop pattern was observed on both sides of lowest point creating an “M shaped” characteristic curve. Similar patterns were observed for both impaired and non-impaired sides across all the subjects where the lowest value represented the IZ location. Both the location and value of the highest and lowest slope varied across all subjects. A two-way SIDE×SLOPE ANOVA analysis revealed significant effects of SLOPE (F_[1,9] _= 24.43, p = 0.001) and SIDE (F_[1,9] _= 8.91, p = 0.015) and a significant SLOPE×SIDE interaction (p < 0.005). Further one-way ANOVA analyses showed that the slopes were significantly greater on the non-impaired side than on the impaired side, and that the highest slope was significantly greater than the lowest slope (Fig. 6).

The highest slope from the linear array recording on each side was selected for further comparison. The correlation coefficients of linear regression were between 0.78 and 0.97 for both sides. On average, slope values were not significantly different between two sides (p = 0.18). However, there was mixed observation of slopes from individual subjects. As shown in Table 1, 4 out of 10 tested subjects had greater slope values on the impaired side.

**Figure 7:**
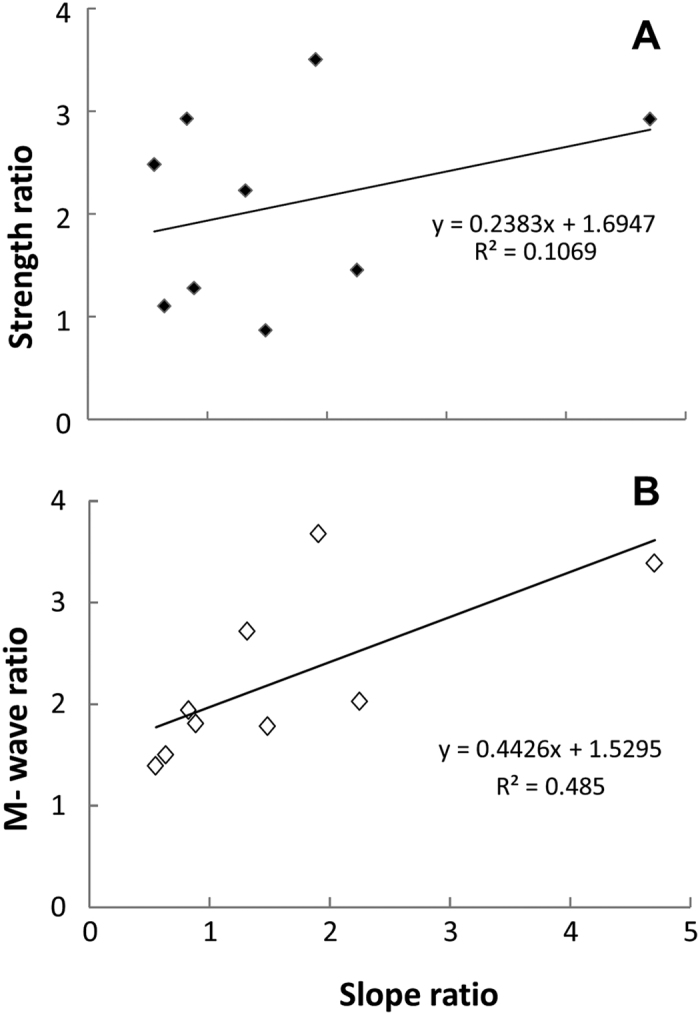
Correlation between strength ratio and slope ratio (upper panel) and between M-wave ratio and slope ratio (lower panel). The ratio was calculated by non-impaired/impaired values. Note: Subject 6 with a slope ratio of 11.3 was excluded from ratio analysis, because the M-wave values were not reliably analyzed for this subject.

Previous studies^7^,^8^ reported large variations in the slope after stroke. To examine the potential effect of weakness after stroke on the EMG-torque relation, linear regression was performed between strength ratio and slope ratio between two sides. As shown in Fig. 7, there was a weak correlation between strength ratio and slope ratio (r = 0.33). The slope ratio range was 1.1 to 4.7. However, there was a moderate to high correlation between M-wave ratio and slope ratio (r = 0.69, see the bottom panel of Fig. 7).

### Innervation zone

**Figure 8:**
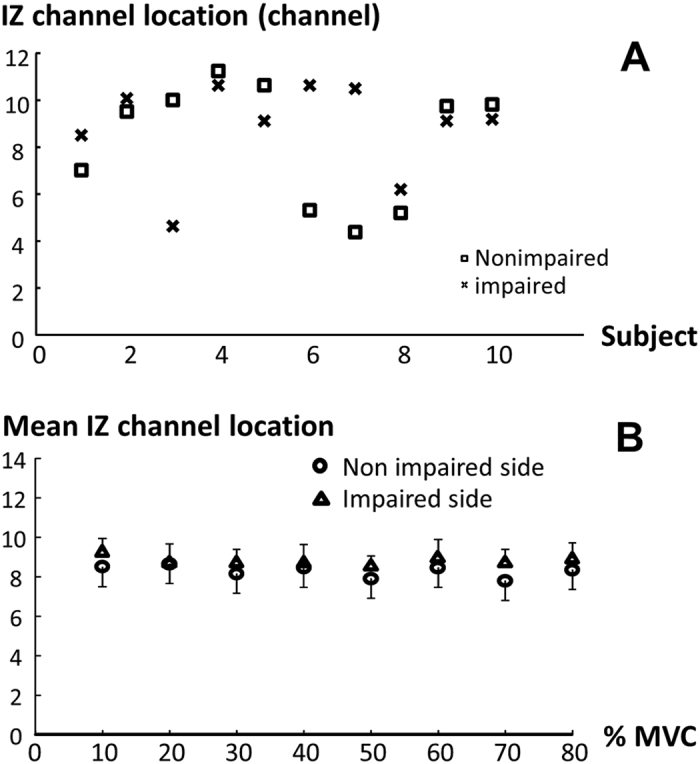
Innervation zone (IZ) channel location variation. (**A**) Asymmetries in IZ channel location between impaired and non-impaired sides at rest. (**B**) Comparison of mean IZ channel location between two sides across all force levels for the whole group. Note that there are individual differences in IZ locations (**A**). However, the intensity of voluntary contraction does not have effect on IZ locations (**B**).

In this study, the IZ location was detected using visual inspection. Even though we carefully placed the linear array symmetrically using anatomical landmarks, IZ location was not symmetrical between the impaired and non-impaired side (Fig. 8A). There were considerable asymmetries in IZ location between two sides in some stroke survivors (Subjects 3, 6, and 7, see Table 1). We then examined whether IZ location determined by visual inspection varied with levels of voluntary activation. As shown in Fig. 8B, IZ location did not vary as a function of voluntary activation. There was no significant change in IZ location with the contraction level on either side. Two way SIDE x ACTIVATION ANOVA analysis established that IZ location showed no significant difference between two sides (main factor of SIDE F_[1,9]_ = 0.59, p = 0.30) and also among contraction level (main factor of ACTIVATION F_[7,63]_ = 1.88, p = 0.08) for the whole group. No significant interactions were found.

## Discussion

In the present study, we re-examined the EMG-elbow flexion torque relation in hemiparetic stroke subjects using a linear electrode array. We confirmed the importance of IZ location on EMG-force relations^27^. We also confirmed that the slope was greater on the impaired side in almost half of the subjects (4 out of 10 subjects) for the range of matched torques on both sides. The main novel findings were that: 1) Asymmetries in IZ location between impaired and non-impaired sides of some stroke survivors; 2) Distribution of the individual channel EMG-torque relation slope showed a quasi-symmetrical “M” shaped pattern with the lowest value corresponding to the IZ location on both the impaired and non-impaired side, and 3) there was a weak correlation between strength ratio and slope ratio of two sides. A moderate to high correlation between the M-wave ratio and the slope ratio was observed in stroke subjects.

### IZ location and EMG-torque relations

The innervation zone from the linear array recording was accurately localized by visual inspection. The IZ location did not show a clear change with the level of elbow voluntary flexion. It has been reported that IZ shifts with changes in joint angle and voluntary activation^28^,^29^,^30^,^31^. In the study by DeFreitas *et al*.^28^, the IZ shifted proximally about 4.5–7.0 mm when isometric elbow flexion torque increased from 20% to 100% MVC. The amount of IZ movement reflected a 2–3 channel shift on a 16 channel array with an inter-electrode interval of 2.5 mm. When a 16 channel linear array with a greater inter-electrode interval (10 mm) was used, Martin and MacIsaac^30^ reported that the IZ location of the biceps brachii muscle was independent of force level. The inter-electrode interval of the linear electrode array in this study was 5 mm. Due to limited resolution, the linear array may be not able to reveal the change of IZ location, if the IZ movement in stroke subjects was similar to the reported ranges in healthy subjects (4.5–7.0 mm^28^, 6.0 mm^29^, and 3.9 mm^31^).

The novel finding of an “M” shaped pattern of the slopes of the individual channel EMG-torque relation further confirmed the relevance of IZ location. This pattern of slope curve indicated the importance of placement of bipolar surface electrodes with respect to the IZ location. Depending on placement of bipolar surface electrode with reference to the IZ location of the individual muscle (Fig. 4), the slope on the impaired side could be greater, similar, or even smaller compared with the non-impaired side, as reported in previous studies^7^,^8^. The importance of electrode placement with respect to IZ location is further signified by asymmetries in IZ location between the impaired and non-impaired sides in some stroke survivors, i.e., IZ location is not certainly based on anatomical symmetry. As shown in Fig. 8A, even when both impaired and non-impaired sides were symmetrically positioned with symmetrical placement of the bipolar surface electrodes following anatomical landmarks, there were considerable asymmetries in some stroke subjects. Asymmetries in IZ location thus undoubtedly contribute to the abnormal EMG-torque relations among stroke subjects. Therefore, comparison of the highest slope value from the linear array recordings between impaired and non-impaired sides has the advantage to minimize or avoid the influence by asymmetrical IZ locations and electrode placement. Such comparison could potentially reflect the true difference between two sides in stroke survivors.

IZ asymmetry also has potential clinical applications. In stroke patients who need Botulinum toxin injection for spasticity management, best clinical outcome could be achieved if the toxins are directly injected to the innervation zones^32^. A small sample size is a limitation of this study. The finding of IZ asymmetry in this study is encouraging, however. Further studies are needed to confirm this finding and to examine whether IZ-guided individualized Botulinum toxin injection could lead to better clinical outcomes.

### Abnormal EMG-torque relations in chronic stroke

Our observation of variable slope values in individual stroke subjects from linear array recordings confirmed findings from the previous studies^7^,^8^, where greater slopes were observed in half of stroke subjects and smaller or similar slopes in the other half. Since the highest slope from the linear array recordings was selected for comparison, this finding of mixed observation suggests that variation in EMG-torque relations in stroke is not only attributable to the effect of bipolar electrode placement or asymmetrical IZ locations. The greater slopes can be due to reduction of motor unit firing rates or muscle fiber re-innervation in paretic muscles as discussed in the Introduction session.

Our findings of a weak correlation between strength ratio and slope ratio suggest that strength is not likely correlated with the EMG-torque relation. As shown in Fig. 3, stroke subjects showed curvilinear EMG-torque relations on the non-impaired side. It increased slowly at the low torques, and more rapidly at high torques. The max strength ratio was 5.8 in this study while 3.3 in the previous study^8^. Given this wide range of strength ratio, we observed a wider range of slope ratio (range of 1.1 to 11.3) in stroke subjects, while the maximum slope ratio was 5.2^7^ or 3.55^8^ in the previous studies (Fig. 7A). In contrast, there was a stronger correlation between M-wave ratio and slope ratio (Fig. 7B). Changes in muscle properties (atrophy, or increased fiber density through re-innervation) could affect M-wave amplitudes. Therefore, the findings of correlation analysis suggest the primary role of peripheral neuromuscular changes in the EMG-torque relation.

Peripheral muscular changes – atrophy and reinnervation both occurred in the impaired muscles^3^,^5^,^33^. The mixed results of greater or smaller slopes in the impaired muscle may reflect different combinations of atrophy and reinnervation in individual muscles. For example, differential loss of large motor units^3^,^4^, recruitment of low-threshold motor units^4^,^5^,^6^,^34^, and muscle fiber atrophy^35^ may account for smaller slopes. On the other hand, increased muscle fiber density through reinnervation and reduction of motor unit firing rates and compression of motor unit recruitment threshold could lead to greater slopes^4^,^5^,^8^,^13^,^36^,^37^,^38^. We acknowledge that a small sample size was a limitation of study. Given heterogeneity of stroke subjects, e.g., large range of ages, we view the high correlation observed in stroke subjects rather positively.

## Conclusion

In summary, re-evaluation of the EMG-elbow flexion torque relation using linear electrode array EMG recordings showed asymmetrical IZ locations and a difference in slope of the EMG-torque relations between impaired and non-impaired sides in stroke survivors. There was a weak correlation between slope ratio and strength ratio, but a stronger correlation between slope ratio and M-wave ratio between two sides of stroke survivors. These findings suggest that the EMG-torque relations are likely mediated and influenced by multiple factors. Possible mechanisms could include weakness and decreased descending activation, altered motor unit control and adaptive peripheral neuromuscular changes, asymmetries in IZ locations, and placement of bipolar electrodes. Our findings emphasize the importance of electrode placement and suggest the primary role of peripheral adaptive changes in the EMG-torque relations after stroke.

## Additional Information

**How to cite this article**: Bhadane, M. *et al*. Re-evaluation of EMG-torque relation in chronic stroke using linear electrode array EMG recordings. *Sci. Rep.* **6**, 28957; doi: 10.1038/srep28957 (2016).

## Figures and Tables

**Table 1:** Subject characteristics and slope of EMG-torque relations (M: male, F: female, age (years), NIP: non-impaired side, IP: impaired side; MAS: modified Ashworth scale; unit for M-wave: uV; for IZ location: channel; for slope: uV/Nm).

ID	Sex	Age	Months after stroke	Impaired side	MAS	MVC (Nm) NIP	MVC (Nm) IP	Slope (NIP)	Slope (IP)	M- wave (NIP)	M- wave (IP)	IZ location (NIP)	IZ location (IP)
1	F	57	68	right	1+	40	18	1.19	0.9	1280	472	7	9
2	M	67	33	right	1+	73	25	1.08	0.23	1314	388	10	10
3	M	61	96	right	0	31	36	3.85	2.59	4586	2576	10	5
4	M	89	74	left	1+	42	12	1.20	0.63	2628	715	11	11
5	M	76	39	right	1	22	20	0.81	1.27	1607	1070	11	9
6	F	60	39	left	0	22	3.8	4.42	0.39	–	–	5	11
7	F	58	19	left	1	19	6.5	2.93	3.54	3975	2052	4	11
8	M	59	68	left	0	58	40	3.94	1.75	4401	2174	5	6
9	M	50	12	right	1	52	21	1.32	2.37	3867	2782	10	9
10	M	47	44	left	0	70	55	3.00	3.38	6265	3464	10	9
